# Inositols: From Established Knowledge to Novel Approaches

**DOI:** 10.3390/ijms221910575

**Published:** 2021-09-30

**Authors:** Simona Dinicola, Vittorio Unfer, Fabio Facchinetti, Christophe O. Soulage, Nicholas D. Greene, Mariano Bizzarri, Antonio Simone Laganà, Shiao-Yng Chan, Arturo Bevilacqua, Lali Pkhaladze, Salvatore Benvenga, Annarita Stringaro, Daniele Barbaro, Marialuisa Appetecchia, Cesare Aragona, Maria Salomè Bezerra Espinola, Tonino Cantelmi, Pietro Cavalli, Tony T. Chiu, Andrew J. Copp, Rosario D’Anna, Didier Dewailly, Cherubino Di Lorenzo, Evanthia Diamanti-Kandarakis, Imelda Hernández Marín, Moshe Hod, Zdravko Kamenov, Eleni Kandaraki, Giovanni Monastra, Mario Montanino Oliva, John E. Nestler, Maurizio Nordio, Ali C. Ozay, Olga Papalou, Giuseppina Porcaro, Nikos Prapas, Scott Roseff, Monica Vazquez-Levin, Ivana Vucenik, Artur Wdowiak

**Affiliations:** 1 Systems Biology Group Lab, 00161 Rome, Italy; simonadinicola.sd@gmail.com (S.D.); vunfer@gmail.com (V.U.); mariano.bizzarri@uniroma1.it (M.B.); aragonacesare@gmail.com (C.A.); espinolasalome@gmail.com (M.S.B.E.); g.monastra@gmail.com (G.M.); 2 Obstetrics and Gynecology Unit, Mother-Infant and Adult Department of Medical and Surgical Sciences, University of Modena and Reggio Emilia, 41121 Modena, Italy; 3 CarMeN Lab, INSA-Lyon, INSERM U1060, INRA, University Claude Bernard Lyon 1, 69100 Villeurbanne, France; christophe.soulage@univ-lyon1.fr; 4 Newlife Birth Defects Research Centre and Developmental Biology and Cancer Programme, Institute of Child Health, University College London, London WC1E 6BT, UK; n.greene@ucl.ac.uk (N.D.G.); a.copp@ucl.ac.uk (A.J.C.); 5 Department of Experimental Medicine, University La Sapienza, 00161 Rome, Italy; 6 Department of Obstetrics and Gynecology, Hospital “Filippo Del Ponte”, University of Insubria, 21100 Varese, Italy; antoniosimone.lagana@uninsubria.it; 7 Department of Obstetrics and Gynecology, Yong Loo Lin School of Medicine, National University of Singapore, Singapore 119228, Singapore; obgchan@nus.edu.sg; 8 Department of Dynamic, Clinical Psychology and Health Studies, Sapienza University, 00161 Rome, Italy; arturo.bevilacqua@uniroma1.it; 9 Zhordania and Khomasuridze Institute of Reproductology, Tbilisi 0112, Georgia; lpkhaladze@yahoo.com; 10 Department of Clinical and Experimental Medicine, University of Messina, 98122 Messina, Italy; s.benvenga@live.it; 11 National Center for Drug Research and Evaluation, Italian National Institute of Health, 00161 Rome, Italy; annarita.stringaro@iss.it; 12 U.O. Endocrinology in Livorno Hospital, USL Nordovest Toscana, 57100 Livorno, Italy; danielebarbaro@katamail.com; 13 Oncological Endocrinology Unit, Regina Elena National Cancer Institute, IRCCS, 00161 Rome, Italy; marialuisa.appetecchia@ifo.gov.it; 14 Institute for Interpersonal Cognitive Therapy, 00100 Rome, Italy; toninocantelmi@tiscali.it; 15 Humanitas Research Hospital, Rozzano, 20089 Milan, Italy; pietro.cavalli@gmail.com; 16 IVF Centre, Hong Kong 999077, China; Tony.Chiu@ivf.hk; 17 Department of Human Pathology, University of Messina, 98122 Messina, Italy; rdanna@unime.it; 18 Faculty of Medicine, University of Lille, 59000 Lille, France; didier.dewailly@orange.fr; 19 Department of Medico-Surgical Sciences and Biotechnologies, Sapienza University of Rome Polo Pontino, 04100 Latina, Italy; cherub@inwind.it; 20 Department of Endocrinology and Diabetes, HYGEIA Hospital, Marousi, 15123 Athens, Greece; e.diamanti.kandarakis@gmail.com (E.D.-K.); elenkand@gmail.com (E.K.); olinpap@hotmail.com (O.P.); 21 Human Reproduction Department, Hospital Juárez de México, Universidad Nacional Autónoma de México (UNAM), Mexico City 07760, Mexico; marime64@hotmail.com; 22 Department of Obstetrics and Gynecology Sackler Faculty of Medicine, Tel-Aviv University, Tel-Aviv 6997801, Israel; hodroyal@inter.net.il; 23 Department of Internal Medicine, Medical University of Sofia, 1431 Sofia, Bulgaria; zkamenov@hotmail.com; 24 Department of Obstetrics and Gynecology, Santo Spirito Hospital, 00193 Rome, Italy; dr.montanino@gmail.com; 25 Division of Endocrinology, Diabetes and Metabolism, Department of Internal Medicine, Virginia Commonwealth University, Richmond, VA 23284, USA; john.nestler@vcuhealth.org; 26 A.S.L. RMF, 00053 Civitavecchia, Italy; maurizionordio1@gmail.com; 27 Department of Obstetrics and Gynecology, Near East University Hospital, Nicosia 99138, Cyprus; alicenk.ozay@neu.edu.tr; 28 Women’s Health Centre, USL UMBRIA 2, 05100 Terni, Italy; giusy.porcaro@gmail.com; 29 IAKENTRO, Infertility Treatment Center, 54250 Thessaloniki, Greece; nikos@iakentro.gr; 30 Reproductive Endocrinology and Infertility, South Florida Institute for Reproductive Medicine (IVFMD), Boca Raton, FL 33458, USA; dr.roseff@ivfmd.com; 31 Instituto de Biología y Medicina Experimental (IBYME, CONICET-FIBYME), Consejo Nacional de Investigaciones Científicas y Técnicas de Argentina (CONICET), Buenos Aires 2490, Argentina; mhvazl@gmail.com; 32 Department of Medical & Research Technology and Pathology, School of Medicine, University of Maryland, Baltimore, MD 21201, USA; ivucenik@som.umaryland.edu; 33 Diagnostic Techniques Unit, Medical University of Lublin, 20-081 Lublin, Poland; wdowiakartur@gmail.com

**Keywords:** myo-Inositol, D-chiro-Inositol, epimerase, FSH, PCOS, steroidogenesis, aromatase, testosterone, GDM, neural tube defects

## Abstract

Myo-inositol (myo-Ins) and D-chiro-inositol (D-chiro-Ins) are natural compounds involved in many biological pathways. Since the discovery of their involvement in endocrine signal transduction, myo-Ins and D-chiro-Ins supplementation has contributed to clinical approaches in ameliorating many gynecological and endocrinological diseases. Currently both myo-Ins and D-chiro-Ins are well-tolerated, effective alternative candidates to the classical insulin sensitizers, and are useful treatments in preventing and treating metabolic and reproductive disorders such as polycystic ovary syndrome (PCOS), gestational diabetes mellitus (GDM), and male fertility disturbances, like sperm abnormalities. Moreover, besides metabolic activity, myo-Ins and D-chiro-Ins deeply influence steroidogenesis, regulating the pools of androgens and estrogens, likely in opposite ways. Given the complexity of inositol-related mechanisms of action, many of their beneficial effects are still under scrutiny. Therefore, continuing research aims to discover new emerging roles and mechanisms that can allow clinicians to tailor inositol therapy and to use it in other medical areas, hitherto unexplored. The present paper outlines the established evidence on inositols and updates on recent research, namely concerning D-chiro-Ins involvement into steroidogenesis. In particular, D-chiro-Ins mediates insulin-induced testosterone biosynthesis from ovarian thecal cells and directly affects synthesis of estrogens by modulating the expression of the aromatase enzyme. Ovaries, as well as other organs and tissues, are characterized by a specific ratio of myo-Ins to D-chiro-Ins, which ensures their healthy state and proper functionality. Altered inositol ratios may account for pathological conditions, causing an imbalance in sex hormones. Such situations usually occur in association with medical conditions, such as PCOS, or as a consequence of some pharmacological treatments. Based on the physiological role of inositols and the pathological implications of altered myo-Ins to D-chiro-Ins ratios, inositol therapy may be designed with two different aims: (1) restoring the inositol physiological ratio; (2) altering the ratio in a controlled way to achieve specific effects.

## 1. Introduction: An Overview on Inositols

### 1.1. Inositol Discovery and Biology

**Figure 1:**
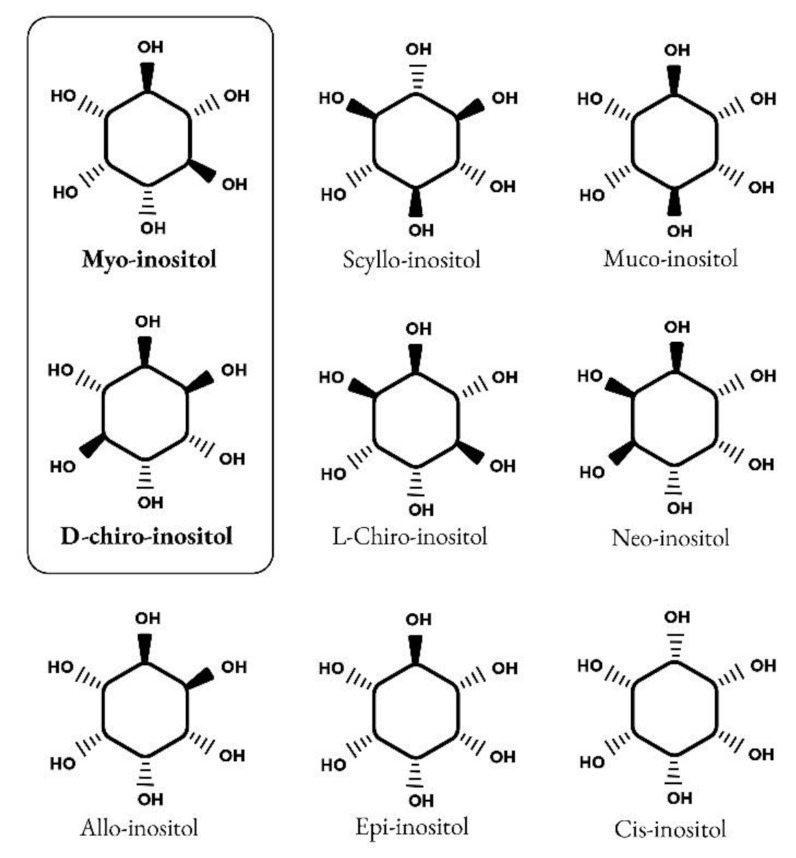
Structure of nine isomers of inositol. Myo-inositol and D-chiro-inositol are the most common isomers of inositol.

Inositols caught the interest of clinicians, especially endocrinologists and gynecologists only in the past twenty years, although their story goes back a long way. It began in 1850 when the German physician and chemist, Johann Joseph Scherer, isolated a hexahydroxycyclohexane from muscle cells and named this molecule “Inositol” from the combination of the Greek terms [ìς (is, in-, “sinew, fiber”), -ose (indicating a carbohydrate), -ite (“ester”), -ol (“an alcohol”)] and also because of its sweet taste [1]. Only many years later, Maquenne established the inositol cyclohexanol structure, purifying it from leaves [2], while a century later the elegant work of Posternak described the configuration of the main inositol isomer in eukaryotic tissues: myo-inositol (myo-Ins) [3]. The structure of this hexahydroxycyclohexane allows the formation of nine different isomers: cis-, epi, allo-, myo-, neo-, scyllo-, L-chiro-, D-chiro- and muco- inositol (Figure 1) [4].

Myo-Ins is an organic osmolyte that regulates cellular responses to hypertonic environments. Although myo-Ins absorption can occur by a diffusion process when it is highly concentrated, inositols uptake by cells is primarily carried out by a complex system of transporters, which mediate an active transport. Na^+^-coupled transport is exerted by sodium/myo-inositol transporter-1 (SMIT1) and sodium/myo-inositol transporter-2 (SMIT2), and H^+^-coupled transport is exerted by H^+^/myo-inositol transporter (HMIT) [5,6,7]. Those Myo-Ins transporters have been found in several tissues including kidney, brain, liver, pancreas, placenta, heart and skeletal muscle [8]. In particular, the expression of SMIT1 is upregulated by extracellular hypertonicity via transcriptional mechanisms, resulting in increased uptake of myo-Ins into cells. This prevents an increase in the concentration of inorganic ions without perturbing the activity of macromolecules [9].

Inositol is an important component of structural lipids, namely phosphatidyl-inositol (PI) and its various phosphates, including phosphatidyl-inositol phosphate (PIP) lipids [10]. Myo-Ins is basically incorporated into eukaryotic cell membranes as phosphatidyl-myo-inositol, the precursor of inositol triphosphate (InsP3), which acts as second messenger in the transduction of several endocrine signals, including FSH, TSH and insulin.

In humans a large amount of myo-Ins (about 1 g/day) is provided by dietary intake, with cereals, legumes, oil seeds and nuts representing the main sources [11], but a significant proportion of daily requirements is still synthesized endogenously (about 4 g/day), with kidneys being the major contributors.

Endogenously, myo-Ins is synthetized from glucose-6-phosphate (G6P), which is isomerized to inositol-3-phosphate (InsP3) by D-3-myo-inositol-phosphate synthase enzyme (inositol synthase, Ino1, or MIPS1) [12]. Then, through inositol monophosphatase-1 (IMPA-1 or IMPase), InsP3 is dephosphorylated into free myo-Ins [13]. Free myo-Ins could also be obtained through dephosphorylation of inositol-1,4,5-trisphosphate (InsP3) and inositol-1,4-bisphosphate (InsP2).

**Figure 2:**
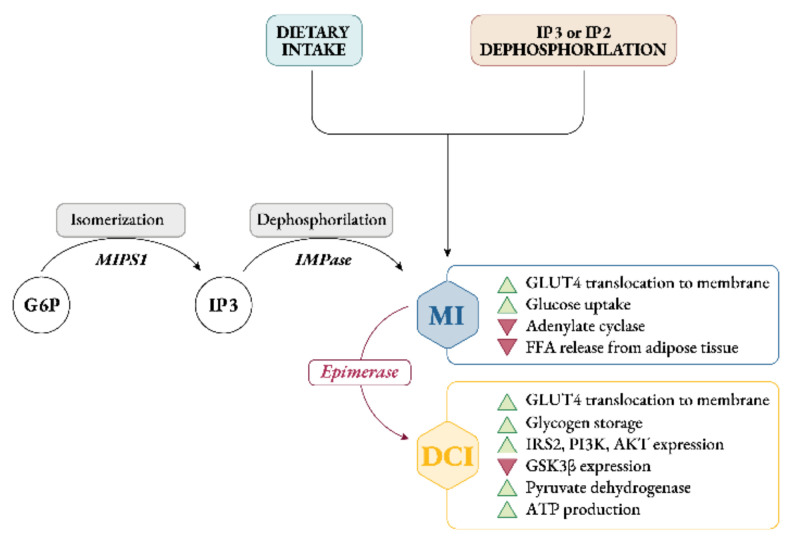
Synthesis, sources and role of myo-inositol and D-chiro-inositol in insulin signaling pathway. Abbreviations: G6P, glucose-6-phosphate; MIPS1, myo-inositol-phosphate synthase; IMPase, inositol monophosphatase; IP3, inositol-trisphosphate; IP2, inositol-biphosphate; MI, myo-inositol; DCI, D-chiro-inositol; GLUT4, glucose transporter type 4; FFA, free fatty acids; IRS2, insulin receptor type 2; PI3K, phosphoinositide 3-kinase; GSK3β, glycogen synthase kinase 3β.

When the endogenous production of myo-Ins is insufficient to meet the human biological needs, an adequate intake of inositol either through specific food and/or supplements becomes necessary [14] (Figure 2).

Intracellularly, myo-Ins promotes GLUT4 translocation to the plasma membrane to enhance glucose uptake, inhibits adenylate cyclase and reduces free fatty acid release from adipose tissue [15,16]. This could explain why tissues with high glucose utilization, like brain, heart and ovary contain a large amount of myo-Ins compared with other tissues [17].

Under insulin stimulation, tissue-specific epimerase enzymes convert myo-Ins into its stereoisomer D-chiro-inositol (D-chiro-Ins) [18]. This is a unidirectional reaction which allows each organ and tissue to benefit from a specific and proper balance between myo-Ins and D-chiro-Ins content, ensuring the correct metabolic functions and consequent physiological status.

D-chiro-Ins stimulates glycogen synthase, and its levels are relatively increased in those tissues involved in glycogen storage such as liver or skeletal muscle [16]. Moreover, D-chiro-Ins increases mRNA and protein expression of IRS2, PI3K and AKT, upregulating the level of the P-AKT protein, and downregulating the level of the GSK3β protein [19] (Figure 2); all of which are key players in insulin and other hormone signal transduction.

By these activities D-chiro-Ins reduces the amount of cytosolic glucose, creating a glucose gradient that facilitates additional uptake of glucose through the mobilization of GLUT4 transporters, which are expressed in intracellular vesicles that are subsequently translocated to the cell membrane [20]. Moreover, D-chiro-Ins is involved in stimulating pyruvate dehydrogenase, which induces glycolysis and the Kreb’s cycle, resulting in the production of adenosine triphosphate (ATP) [21,22].

Through all these mechanisms myo-Ins and D-chiro-Ins may exert their insulin-sensitizing effect thereby decreasing insulin requirements, which are consequently reflected by lower circulating insulin concentrations [23].

**Figure 3:**
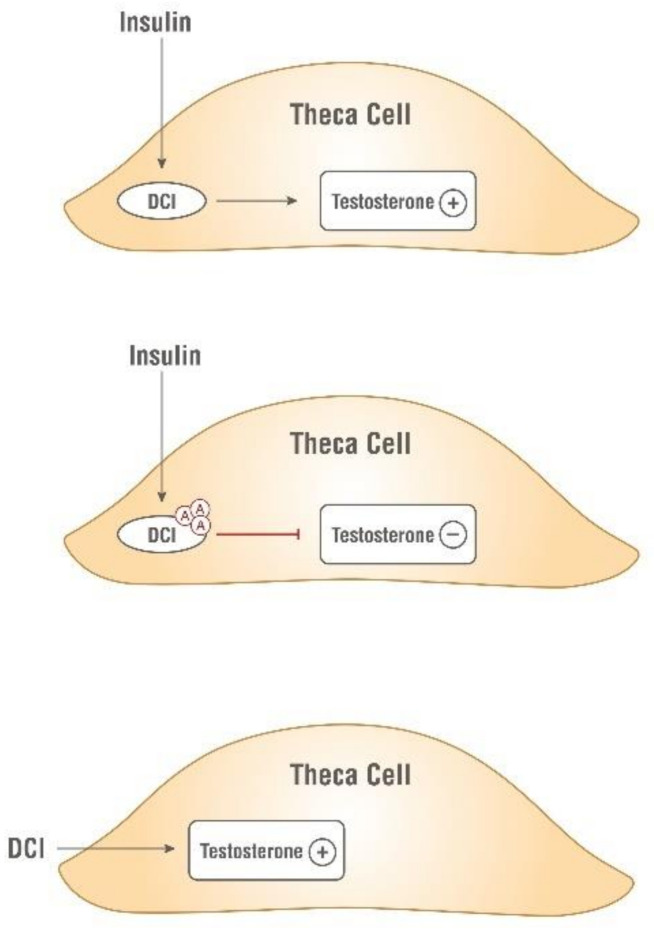
Insulin-mimetic action of D-chiro-Ins (DCI). Both insulin and D-chiro-Ins stimulate testosterone production by theca cell. Antibodies, Ⓐ, against this glycan block testosterone production.

Prof. Larner and colleagues were the first to propose inositols as chemical insulin mediators [22,24,25]. Their group isolated and purified two glycans containing D-chiro-Ins and myo-Ins, respectively. Both glycans showed insulin mimetic properties when administered in vivo. Indeed, when injected intravenously, these compounds dose-dependently reduced hyperglycemia in streptozotocin-induced diabetic rats, while when injected intraperitoneally, they stimulated labeled glucose incorporation into glycogen in rat diaphragm muscle [26]. Independently, Nestler and coworkers confirmed the insulin-mimetic action of a glycan containing D-chiro-Ins (called INS-2) on human ovarian thecal cells [27,28]. Nestler et al. observed that both insulin and D-chiro-Ins stimulated the biosynthesis of testosterone which was blocked by an antibody directed against this glycan (Figure 3).

These findings raised the interest of the scientific community in inositols’ insulin-mimetic properties, and the investigation of their usefulness in clinical practice increased concomitantly.

### 1.2. Inositols and Human Reproduction

**Figure 4:**
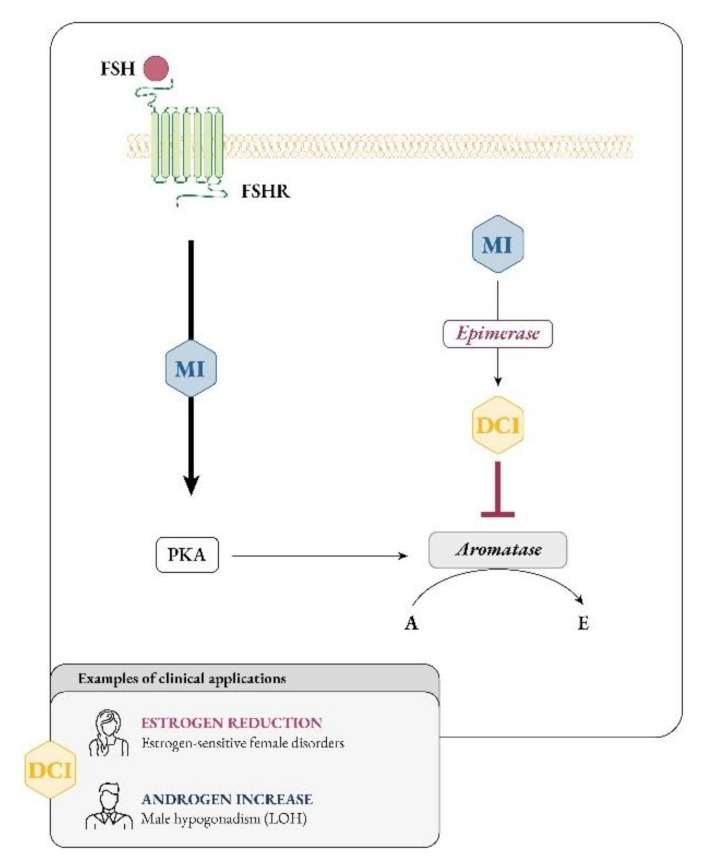
Myo-inositol and D-chiro-inositol affect aromatase activity in an opposite manner. Abbreviations: FSH, follicular stimulating hormone; FSHR, FSH receptor; MI, myo-inositol; DCI, D-chiro-inositol; PKA, protein kinase A; A, androgen; E, estrogen; LOH, late onset hypogonadism.

It soon became clear that besides glucose metabolism, inositols are deeply involved in the physiology of female and male reproduction. In women, inositol, specifically myo-Ins, acts as an FSH second messenger (Figure 4) and is involved in FSH-mediated pathways that regulate proliferation and maturation of granulosa cells. Based on this role, myo-Ins modulates the FSH-mediated anti-Mullerian hormone (AMH) production, playing a pivotal role in determining oocyte maturation and transport in the oviduct as well as ensuring the good quality of embryos [29].

Ovaries, as well as other organs and tissues, are characterized by a specific ratio of myo-Ins to D-chiro-Ins, which ensures their healthy state and proper functionality (Table 1). In agreement with its functions, it can be guessed that the concentration of myo-Ins in a healthy female reproductive tract should be higher than that of D-chiro-Ins, supporting its important role in the ovaries. On the other hand, high D-chiro-Ins levels can negatively impact on the quality of oocytes and blastocysts [30], therefore its abundance needs to be tightly regulated.

The impact of inositols on reproductive physiology must consider another inositol effect, namely, their influence on steroidogenesis, though this remains a poorly explored pathway. Indeed, both myo-Ins and D-chiro-Ins deeply affect the androgenic and estrogenic pools, likely in opposite directions.

Larner and Nestler mostly focused on D-chiro-Ins, concluding that this specific inositol influences steroidogenesis stimulating the ovarian production of androgens by thecal cells. Recently, Sacchi and coworkers proposed a second mechanism by which D-chiro-Ins could influence steroidogenesis, namely, by modulating the expression of aromatase enzyme and directly downregulating the synthesis of estrogens [31]. Considering that the healthy physiological status of tissues depends on a proper ratio of inositol concentrations, it is likely that an alteration of myo-Ins to D-chiro-Ins ratio may explain the imbalance in sex hormones observed in some pathological conditions, such as polycystic ovarian syndrome (PCOS), or secondary to pharmacological treatments, malabsorption or competition with glucose in food and beverages.

Overall, considering the growing interest in the clinical use of inositols, and based on the new findings on D-chiro-Ins activity, the present position paper aims to gather the most established evidence on the use of inositols in clinical practice as well as to introduce some novel therapeutic approaches, to allow clinicians to tailor inositol therapy for use in other medical areas, hitherto unexplored.

A non-systematic review was done through a search on the following databases: MEDLINE, EMBASE, Global Health, The Cochrane Library (Cochrane Database of Systematic Reviews, Cochrane Central Register of Controlled Trials, Cochrane Methodology Register), Health Technology Assessment Database and Web of Science.

We selected papers written in English, with no time restrictions about the year of publication. The keywords used for this search were: myo-inositol; D-chiro-inositol; epimerase; FSH; PCOS; steroidogenesis; aromatase; testosterone; GDM and neural tube defects.

Titles and/or abstracts of studies retrieved using the search strategy, and those from additional sources, were screened independently by two review authors to identify studies that potentially meet the aims of this non-systematic review. The full text of these potentially eligible articles was retrieved and independently assessed for eligibility by another two review team members. Any disagreement between them over the eligibility of articles was resolved through discussion with a third (external) collaborator. Two authors independently extracted data from articles about study features and included populations, type of intervention and outcomes. Any discrepancies were identified and resolved through discussion (with a third external collaborator where necessary). Due to the nature of the findings, we opted for a narrative synthesis of the results from selected articles.

According to the physiological role of inositols and the pathological implications of altered myo-Ins to D-chiro-Ins ratios, inositol therapy may be designed with two different aims: restoring an inositol physiological ratio or altering this ratio in a controlled manner to achieve specific effects.

## 2. Evidence-Based Effects of Inositols Supplementation on Human Reproduction

### 2.1. Inositol Supplementation and Female Reproduction (PCOS Metabolism and Ovulation)

PCOS is the most prevalent endocrine disorder in women of reproductive age. According to the Rotterdam criteria, its current definition requires at least two of the following clinical manifestations: chronic ovulatory disorder (oligo-ovulation to anovulation, and amenorrhea), presence of polycystic ovaries at the ultrasound examination, and hyperandrogenism [33].

Insulin resistance and compensatory hyperinsulinemia seem to have a central role in PCOS pathogenesis [34,35], contributing both directly and indirectly to hyperandrogenism development and related clinical features. Indeed, insulin directly stimulates the ovarian theca cells to produce increased levels of androgens and inhibits hepatic synthesis of sex hormone-binding globulin (SHBG), thus indirectly increasing circulating free androgens.

The effectiveness of insulin-sensitizing drugs, such as metformin and thiazolidinediones, in improving ovulatory function and reducing androgen excess in PCOS patients provided additional evidence to support the pathogenic role of insulin resistance in PCOS patients [36].

In this context, inositols, for their metabolic and hormonal functions, can be proposed as effective and well-tolerated alternative approaches to these drugs.

**Figure 5:**
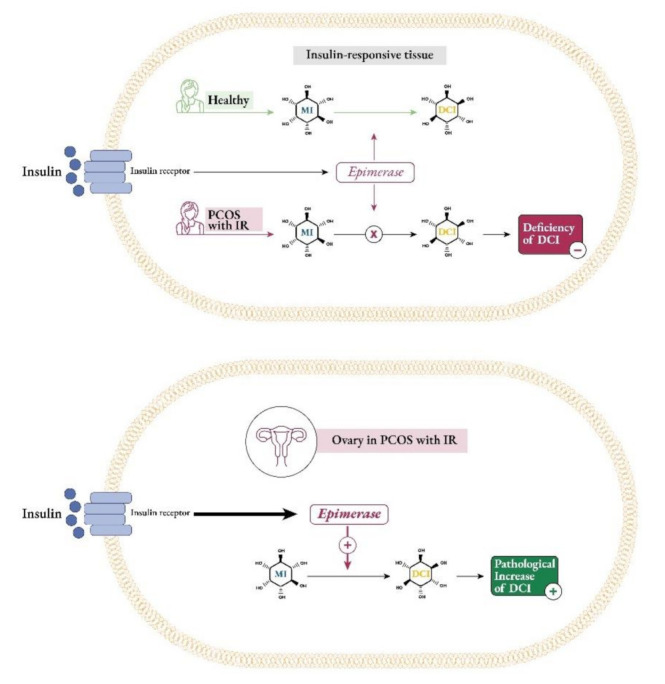
Insulin stimulates epimerase enzyme to convert myo-inositol (MI) to D-chiro-inositol (DCI). The classic insulin target tissues of PCOS women with insulin resistance (IR) show less epimerase activity and consequently result in a systemic deficiency of DCI. Ovaries of PCOS women with IR maintain the normal insulin sensitivity (“ovarian paradox”); the hyperinsulinemia overstimulates epimerase to convert MI to DCI, resulting in a pathological increase in DCI.

In 2013, an International Consensus Conference on myo-Ins and D-chiro-Ins in Obstetrics and Gynecology recognized that both inositols are involved in PCOS pathogenesis and a compelling body of clinical evidence demonstrated that their supplementation could be beneficial in improving metabolic and reproductive features [37]. Notably, pathological conditions, like type 2 diabetes, show decreased insulin sensitivity in many tissues, leading to reduced epimerase activity and lower D-chiro-Ins production [18,38]. However, unlike most tissues, ovaries can maintain normal insulin sensitivity, despite the presence of insulin resistance. Indeed, according to the so-called “ovarian paradox”, ovaries never become insulin resistant, and therefore, the compensatory hyperinsulinemia overstimulates ovarian epimerase activity, causing excessive D-chiro-Ins synthesis at the expense of myo-Ins concentration [39,40] (Figure 5).

The resultant impaired ovarian myo-Ins to D-chiro-Ins ratio may account for PCOS pathogenesis in insulin resistant patients. In fact, the increase in D-chiro-Ins concentration promotes androgen synthesis, meanwhile myo-Ins depletion worsens FSH signaling and oocyte quality [21,40]. Both studies led to similar outcomes, namely that healthy women’s ovaries showed higher myo-Ins levels and lower concentrations of D-chiro-Ins (with a ratio around 100:1); on the contrary, ovaries in PCOS patients proved to have marked myo-Ins depletion and increased D-chiro-Ins content (with a ratio dropping to 0.2:1).

This “ovarian paradox hypothesis” may help to explain why supplementation with D-chiro-Ins alone, especially at high doses (>1200 mg) and for a prolonged time (>3 months), cannot be considered an effective approach to manage PCOS, despite some encouraging results in 1999 [41]. Indeed, in that study the administration of 1200 mg/day of D-chiro-Ins for six to eight weeks to obese, hyperinsulinemic PCOS women reduced serum testosterone level and improved both their ovulatory function and metabolic parameters. In such cases, D-chiro-Ins treatment presumably reduced systemic insulin levels, leading to an increase in intraovarian myo-Ins, which improved FSH sensitivity and restored ovulation in the short term. The same group tried to reproduce their findings supplementing PCOS women with 2400 mg of D-chiro-Ins for 6 weeks [42]. The authors found that, while insulin sensitivity and metabolic parameters significantly improved, testosterone levels did not show the same trend as previously observed. Indeed, after treatment with D-chiro-ins, testosterone did not decrease, showing instead a non-significant increase in average levels (total testosterone +14.7% versus basal level; free testosterone +29%).

When D-chiro-Ins dosage was further increased up to 2400 mg/day, the improvements in the metabolic and hormonal picture found in Nestler’s first study and Cheang’s failed to be confirmed, underlying that crucial differences may emerge when different D-chiro-Ins dosages are used.

In contrast, several lines of evidence proved myo-Ins efficacy and safety in managing PCOS symptoms and improving outcomes [43], with the most promising clinical results observed in obese, insulin-resistant PCOS women, when combining myo-Ins and D-chiro-Ins in a 40:1 ratio [44,45,46,47,48,49,50]. Although other ratios were analyzed, these combinations were without a scientific rationale [51]; conversely the 40:1 ratio that may appear arbitrary, actually is similar to the plasma ratio reported in healthy women [51,52,53,54], thus supporting its supplementation to restore the physiological concentrations of myo-Ins and D-chiro-Ins. On the other hand, one study [55] considers the amount of DCI in the 40:1 ratio very low and proposes combining the two inositols using D-chiro-Ins at high doses. However, some concerns arise when D-chiro-Ins is used at high doses and for prolonged time, as demonstrated in a recent paper by Bevilacqua et al. [56] and discussed below.

### 2.2. Pregnancy Development (NTDs in Folic Acid Resistant Women, GDM Prophylaxis)

Increased insulin resistance and hyperglycemia occurring during pregnancy configure a clinical picture of gestational diabetes mellitus (GDM). This condition may predispose the mother to several risks such as gestational hypertension, cesarean section, and later development of type 2 diabetes, and the baby to preterm birth, congenital abnormalities, macrosomia, being large for gestational age (LGA), neonatal hypoglycemia and respiratory distress syndrome [57]. Furthermore, infants delivered by women with a GDM diagnosis show an increased risk of impaired glucose regulation in later life, further extending the intergenerational cycle of obesity and diabetes [58]. Therefore, the screening for GDM should be universal [59] and ideally conducted between 24–28 weeks of gestation. In high-risk patients, earlier screening for impaired glucose regulation should be strongly considered.

As more evidence arises, continual updating of clinical guidelines for GDM prevention/treatment is needed. Present scientific literature provides essentially three broad approaches to manage GDM: lifestyle changes (diet and exercise), treatment with metformin or insulin (pharmaceutical) and inositol supplementation (nutraceutical). An adequate-diet quality and exercise are the first line therapies to normalize glycemia. However, when adherence to lifestyle changes is poor or glycemic targets cannot be achieved, an intervention is advisable. Metformin use in pregnancy is rapidly increasing, especially for its ability to cross the placenta [60,61,62]. However, evidence fails to support other beneficial effects for this molecule on top of promoting euglycemia [63,64]. Indeed, for the purposes of GDM prevention, earlier observational studies [65,66,67] suggested that metformin administration could be associated with lower risk of GDM development, but these results were not confirmed by subsequent trials [68]. Moreover, despite metformin being generally considered safe during pregnancy, long-term safety data in the offspring is still lacking [60,61,62].

Conversely, several clinical studies have demonstrated the effectiveness and tolerability of inositols, mainly myo-Ins, in GDM prevention and treatment. 

Two recent reviews summarized these trials, concluding that myo-Ins, rather than D-chiro-Ins or their combination, can lower GDM rates, improve gestational glycemia and lipid and insulin resistance parameters as well as reducing the need for insulin therapy should GDM develop later [69,70]. Furthermore, other more recent independent reviews confirmed the potential of myo-Ins supplementation in preventing GDM in selected groups of women [71,72,73].

Finally, interesting results were derived from the latest study by D’Anna and colleagues on GDM treatment [74]. The authors enrolled 120 patients with a diagnosis of GDM and randomized them to two groups: 60 women (treated group) were supplemented with 2 g myo-Ins plus 50 mg α-lactalbumin (α-LA) and 200 µg folic acid (FA) twice a day, whereas another 60 women (control group) received 200 µg FA twice a day. After two months, women in the treated group taking the combination of myo-Ins and α-LA showed reduced insulin resistance, proportion of women requiring insulin, fetal abdominal circumference and neonatal subcutaneous adipose tissue thickness compared to the control group. Moreover, no cases of pre-term birth occurred in the treated group, compared with 15.2% in the control group.

Almost simultaneously, the NiPPeR international multicenter double-blind randomized controlled trial was published [75], acting as an outside voice among the previous studies. In this study the authors investigated the effect on gestational glycemia of a nutritional formulation containing myo-Ins, probiotics, and multiple micronutrients taken preconceptionally and throughout pregnancy. The results recorded by previous studies were not replicated, as there was no significant difference in gestational glycemia, the incidence of GDM and LGA infants. The authors suggested that this complex formulation failed to lower glycemia, due to the design of the study that recruited generally healthy women (many of whom were not at increased risk of GDM), commenced the intervention before pregnancy (instead of starting in the later part of the first trimester of pregnancy) and the possibility of counter-effects by other components of the supplement. It is also the only trial that had a substantial proportion of non-white women, particularly Chinese and other Asian ethnicities.

It is possible that myo-Ins taken alone may have had an effect more consistent with previous studies.

Overall, despite the encouraging findings on different categories of women at risk, namely overweight, obese, and those with PCOS, there is still inadequate evidence for routine myo-Ins supplementation to be included as recommendations in current clinical guidelines for GDM management. Anyway, considering the general agreement among a body of experts that myo-Ins administered in early pregnancy effectively prevents GDM onset, incorporation of such recommendations could happen soon.

Inositol status appears to be associated with susceptibility to neural tube defects (NTDs), a group of congenital malformations affecting the developing brain and spinal cord [76]. Evidence suggests a direct requirement for inositol in neural tube closure, while impaired inositol status is implicated in the potential mechanism by which maternal diabetes is associated with NTDs.

A direct requirement for inositol in neural tube closure was shown in rodents, in which inositol deficiency in whole embryo culture resulted in cranial NTDs [77,78]. In humans, an association of low maternal serum myo-Ins levels with occurrence of spina bifida was identified [79], suggesting a possible link between insufficient inositol and NTDs as reported in rodent models. It has been hypothesized that genetic defects of myo-inositol synthase ISYNA1, which is also expressed in placenta and yolk sac [80,81], may result in low maternal and/or embryonic intracellular myo-Ins, predisposing to NTD pathogenesis. However, a case-control trial study did not confirm this correlation [82]. On the other hand, during the early weeks of human pregnancy inositol concentration in the embryonic compartment is already significantly higher than in maternal serum [83]. This suggests that active, carrier-mediated transport mechanisms for inositol are established early in pregnancy and/or there is substantial placental/fetal production of inositol [84].

**Figure 6:**
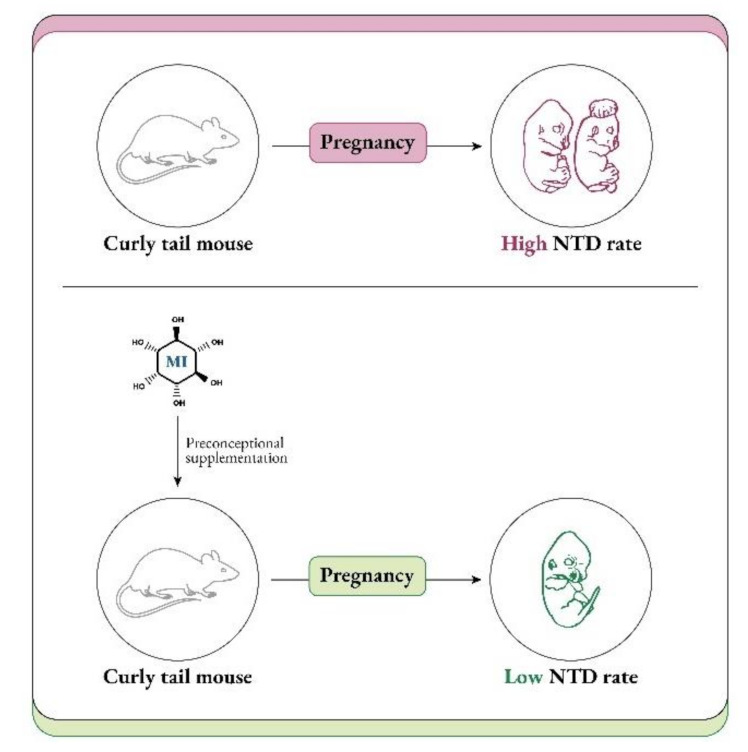
The curly tail model is genetically not responsive to preventive folic acid, exposing pregnant mice to a high neural tube defect (NTD) rate. Preconception supplementation with myo-inositol (MI) may prevent NTD occurrence, lowering the NTD rate.

Evidence for a potential protective effect of inositol for spinal NTDs comes from studies in the curly tail mouse strain, in which NTDs are not preventable by FA, modelling FA-resistant NTDs in humans [85]. Inositol supplementation reduces the incidence of spina bifida in these mice (Figure 6), whether by maternal supplementation or in cultured embryos [86]. Indeed, both myo-Ins and D-chiro-Ins can prevent NTDs in the curly tail model, with the involvement of specific isoforms of protein kinase C [87,88].

The high myo-Ins content in the developing central nervous system, skeletal, and cardiac muscle emphasizes the high embryonic demand for this nutrient [89]. Myo-Ins acts as a precursor for the generation of phosphoinositides that mediate intracellular signaling [76,90]. Myo-Ins, as well as other polyols, may have multiple other functions during embryogenesis, acting as osmolytes to promote expansion of the amniotic and coelomic cavities, providing precursors of cell membrane components and supplying substrates for the pentose phosphate pathway necessary for the synthesis of nucleic acids [83]. Moreover, the high concentration of polyols in the embryonic compartment may reflect an early dependence on the polyol metabolic pathways that serve to maintain ATP concentrations and redox potential while the embryo develops in a low oxygen environment [83].

In addition to the direct requirement for myo-Ins in formation of the neural tube, sub-optimal inositol status may also play a role in the known association of maternal diabetes and obesity with NTDs [91,92]. Although the precise mechanisms leading to diabetes or obesity related NTDs are still unclear, it is likely that a dysregulation in glucose metabolism may be involved. As such, hyperinsulinemia has been proposed as a putative mechanism which determines NTD risk in obese women with diabetes [93]. Moreover, NTDs have been significantly correlated with an increased sugar intake by mothers, even if they did not suffer from overt diabetes, and with maternal features of metabolic syndrome [94,95,96].

Diabetic tissues are prone to be inositol-deficient and show high glucose concentration [97] and inositol depletion, as well as NTDs, are also found in rodent embryos exposed to hyperglycemia or induced diabetes [98,99]. In these models, supplementation with myo-Ins restored myo-Ins tissue content and reduced the incidence of NTDs, suggesting the involvement of lowered myo-Ins in the mechanism of diabetic embryopathy [100,101].

Overall, susceptibility to NTDs is thought to be influenced by the interplay between genetic risk factors and environmental factors, including maternal nutrition [102]. There is compelling evidence that sub-optimal maternal blood folate concentrations can predispose to NTDs and maternal supplementation with folic acid in the periconceptional period, via supplements or food fortification, significantly reduces this risk [103,104,105]. However, FA alone does not provide complete prevention of recurrence or occurrence of NTDs, leading to the description of ‘folic acid resistant NTDs’ [106].

Owing to the prevention of NTDs in the FA-resistant curly tail mouse model, 12 women with a history of FA-resistant NTDs, were counselled to take supplemental myo-Ins as a possible means to prevent NTD recurrence in a subsequent pregnancy [107,108]. All the babies were born without NTDs, which encouraged the view that larger, randomized studies should be performed. A phase I/II double-blind, case-control clinical trial (the PONTI study) recruited women with one or more previous NTD pregnancies who were planning to become pregnant again [109]. Forty-seven participants were randomized to receive a periconception daily supplement containing 1 g myo-Ins plus 5 mg FA, or placebo plus FA. In addition, further non-randomized women were followed up. Overall, no NTDs recurred among the 35 myo-Ins supplemented pregnancies compared with three NTDs among the 22 pregnancies in which only FA supplements were used. Collectively, these studies strongly suggest the potential benefit of combined supplementation with myo-Ins and FA in the periconceptional period, particularly for those women with previous FA-resistant pregnancies. However, considering the good safety profile of the myo-Ins molecule, the use of myo-Ins supplements together with FA, could be recommended for every woman at high risk for NTDs. Furthermore, the findings from clinical studies to date support the need for a further fully powered clinical trial to definitively confirm whether combined myo-Ins and FA supplementation prevents more NTDs than FA alone.

### 2.3. Male Reproduction (Impact on Sperm Parameters)

As observed in females, in male reproductive organs myo-Ins is more abundant than in the bloodstream, indicating that this compound also exhibits a pivotal function in male reproduction. Indeed, transgenic mice with low myo-Ins in their epididymis showed a reduced fertility [110]. In spermatozoa, myo-Ins behaves as second messenger modulating the intracellular Ca^2+^ levels that regulate mitochondrial oxidative metabolism and ATP production [111].

Since they represent a source of energy, mitochondria are key organelles for sperm motility, acrosome reaction and fertilization [111]. Therefore, a healthy mitochondrial state and consequently a high mitochondrial membrane potential (MMP) are considered important features and/or advisable treatment outcomes for good quality sperm.

As myo-Ins is involved in regulating sperm motility, capacitation and acrosome reaction of sperm cells [112], it was successfully used to treat men with fertility dysfunction, especially oligoasthenoteratozoospermia (OAT), a disorder resulting from low sperm count (oligozoospermia), reduced sperm motility (asthenozoospermia) and morphological abnormalities in sperm cells (teratozoospermia).

Studying the spermatozoa from patients with OAT found that inositol monophosphatase-1 (IMPA-1) enzyme was overexpressed, dysregulating the phosphatidyl inositol signaling cascade. Furthermore, these cells exhibited reduced motility [113], suggesting that inositol pathways are crucial to support sperm cell movement.

An in vitro study correlated the low sperm mobility with the presence of an amorphous fibrous material covering the spermatozoa of men with OAT, resulting in an increase in their seminal fluid viscosity. In addition, mitochondrial cristae in the intermediate tract of these spermatozoa were damaged. Incubation with myo-Ins dissolved the amorphous fibrous material and reduced damaged cristae. Moreover, myo-Ins increased MMP and improved sperm parameters including motility and fertilization capacity [112,114].

However, some patients could not benefit from the administration of myo-Ins alone, in terms of increased sperm motility. In this regard, Condorelli et al. [115] observed that D-chiro-Ins in vitro ameliorates the sperm mitochondrial function, evaluated in terms of increased MMP. Indeed, both in case of normozoospermic and asthenozoospermic samples, D-chiro-Ins decreased the percentage of spermatozoa with low MMP in a dose-dependent manner, while increasing the percentage of spermatozoa with high MMP. This pilot study, even though suggesting a better quality for D-chiro-Ins treated spermatozoa, has some limitations. In particular, as evidenced by the authors themselves, MMP is just a predictive parameter of suitable motility and cannot be representative of the overall mitochondrial function. Moreover, an excessively high MMP value might worsen sperm motility and quality, leading to an increased production of reactive oxygen species (ROS) [115]. Several studies on patients undergoing in vitro fertilization (IVF) procedures reported that after incubation with myo-Ins sperm cell count and motility improved as well as the percentage of fertilization increased, both in normal and OAT patients [116,117,118,119]. Besides improving semen characteristics, myo-Ins administration also rebalanced the levels of key reproductive hormones, such as LH, FSH and Inhibin-B [120].

Further interesting results came from the studies carried out by Montanino Oliva and colleagues. In the first one [121], the authors supplemented men with metabolic syndrome and low sperm mobility with a mix of myo-Ins, L-carnitine, L-arginine, vitamin E, Selenium and folic acid twice daily. After 3 months of this treatment, they observed beneficial effects on insulin sensitivity and semen parameters. Indeed, abnormalities in sperm concentration, motility and morphology were significantly ameliorated.

In the second study Montanino Oliva [122] evaluated myo-Ins impact on sperm motility in vagina. Eighty-six couples were enrolled: 43 were treated with vaginal suppositories containing myo-Ins, while another 43 couples, representing the control group, received placebo suppositories. The authors concluded that myo-Ins treatment improved the total sperm motility (54.42% ± 8.72) in respect to baseline value (46.48% ± 4.05) and to placebo group (46.21% ± 5.33). Moreover, myo-Ins treatment increased the percentage of spermatozoa with progressive motility, considered the best marker to identify good quality and healthy sperm cells and essential when pregnancy is the aim of a sexual intercourse. Interestingly, treatment with myo-Ins had a beneficial impact not only on semen parameters but also on cervical mucus quality, reducing its viscosity. As a result, 18.60% of treated couples achieved a pregnancy compared to only 6.97% in the control group.

Recently, the efficacy of myo-Ins supplementation to men with OAT was confirmed and supported by Santoro et al. [123]. In their study the authors investigated, in vitro and in vivo, several sperm parameters, such as motility, survival and capacitation, as well as glucose and lipid metabolisms. They concluded that besides increased motility and improved sperm survival, myo-Ins positively influenced glucose and lipid metabolisms, improving sperm performance.

Lastly, it should be mentioned that myo-Ins is also able to protect the sperm of infertile men undergoing assisted reproductive technology (ART) from alterations occurring during the cryopreservation procedure [124,125]. Indeed, myo-Ins supplementation could significantly increase the cryo-survival rate in samples with abnormal pre-freeze semen characteristics. Taken together, these findings remark once again the beneficial effect of this inositol on human fertility.

## 3. Emerging Roles for Inositol Treatments

### 3.1. The Importance of Myo- to D-chiro-Inositol Ratio in Steroidogenesis

As research progresses, it is becoming clearer that, besides constituting the intracellular second messengers of insulin signaling, inositols also function as endocrine modulators, influencing steroidogenesis. In 1998 Nestler first observed that D-chiro-Ins increased testosterone levels in theca cells from women with PCOS, even though the mechanism underlying this effect remained unknown [28]. Recently, new experiments suggested that D-chiro-Ins directly regulates the gene expression of enzymes involved in steroidogenesis in human granulosa cells, dose-dependently reducing the expression of both aromatase and cytochrome P450 side-chain cleavage genes [31]. Therefore, D-chiro-Ins modulates estrogen levels without completely blocking their biosynthesis.

On the other hand, evidence on the effect of myo-Ins is still needed and we speculate that this isomer may affect aromatase activity in an opposite manner from D-chiro-Ins. In support to this idea, myo-Ins is involved in modulating FSH signaling, and FSH stimulates aromatase synthesis, a fundamental step for conversion of androgens to estrogens and for follicle maturation [126]. FSH downregulation and the subsequent decrease in aromatase synthetized by granulosa cells constitute a hallmark of PCOS [127]. Thus, while D-chiro-Ins inhibits aromatase, myo-Ins seems to enhance aromatase synthesis in granulosa cells [128,129]. Moreover, myo-Ins could modulate ovarian steroidogenesis by rearranging cytoskeletal proteins [130].

In this regard, higher myo-Ins/D-chiro-Ins ratios should increase aromatase activity in granulosa, fostering estrogen biosynthesis, while lower myo-Ins/D-chiro-Ins ratios promote androgen production in thecal cells [131]. 

As a matter of fact, under normal homeostatic conditions, ovarian myo-Ins/D-chiro-Ins ratio ranges from 70:1 to 100:1, while in PCOS women this ratio decreased pathologically in favor of D-chiro-Ins [21,40]. The increased D-chiro-Ins concentration promotes androgen synthesis, while myo-Ins depletion worsens the energy state of the oocytes, impairing FSH signaling and oocyte quality [30].

Bevilacqua et al. contributed to the first demonstration in a pre-clinical model of how the myo-Ins to D-chiro-Ins ratio influences ovarian physiology [46]. In this study the authors investigated supplementation with different myo-Ins to D-chiro-Ins ratios (namely 5:1; 20:1; 40:1 and 80:1) for their ability to revert the PCOS phenotype back to normal. Among the various ratios, the 40:1 reached the most significant results. Indeed, mice supplemented with this myo-Ins/D-chiro-Ins ratio rapidly and almost completely recovered from PCOS signs and symptoms. The other ratios were less effective or even produced detrimental effects, especially with the formulation containing the highest D-chiro-Ins content.

These results were confirmed in a clinical trial by Nordio et al. [44]. In this study fifty-six patients with PCOS were supplemented with different myo-Ins/D-chiro-Ins ratios (namely 0:1; 1:3.5; 2.5:1; 5:1; 20:1; 40:1 and 80:1) with the aim to restore ovulatory function, as evidenced by progesterone assay, and ameliorate abnormalities in metabolic parameters, like FSH, LH, SHBG, E2, free testosterone, HOMA index and basal and postprandial insulin. The authors observed that the 40:1 combination produced the most significant improvements, followed by the 20:1 and 80:1, while the other ratios showed less relevant outcomes. Despite these promising results, of course, future studies are mandatory to shed light on the molecular aspects of ovarian inositol activity and to investigate the beneficial effects of an ideal myo-Ins/D-chiro-Ins formulation in larger cohorts of patients, possibly with different PCOS phenotypes.

### 3.2. Myo-Inositol Support to Assisted Reproduction Techniques (ART)

In 1992 Chiu and colleagues first demonstrated the beneficial role for myo-Ins in IVF treatment [132]. They found that patients who successfully conceived with IVF showed a higher level of this inositol in their serum (the day before hCG injection) and postulated that myo-Ins could participate in facilitating the early phases of IVF as well as in proper embryonic development.

Ten years later the same group discovered that a correlation exists between myo-Ins concentration in follicular fluid (FF) and oocyte quality [133]. Indeed, they observed that follicles with higher levels of myo-Ins contained good quality oocytes, implying a potential association between myo-Ins and oocyte maturation, with the prospective of using myo-Ins as a marker of oocyte quality. These findings conflicted with Nestler’s theory that hypothesized a decreased myo-Ins to D-chiro-Ins epimerase activity as a crucial factor in PCOS pathogenesis. Indeed, according to Nestler’s theory, the supplementation of myo-Ins would be expected to be ineffective in PCOS patients.

The correlation between the levels of myo-Ins and D-chiro-Ins in FF and the blastocyst quality only received a clearer elucidation with the study of Ravanos and coworkers [30]. They observed that blastocysts showing good quality were associated with a higher myo-Ins to D-chiro-Ins ratio in FF, compared to those rated as poor-quality that exhibited a large amount of D-chiro-Ins. This striking result suggests that the ratio between myo-Ins and D-chiro-Ins may be a valuable biomarker for blastocyst quality, especially in forecasting achievement of pregnancy.

From this evidence, the use of myo-Ins to support ART in PCOS women has figured prominently in scientific literature, since PCOS patients often experience infertility. In a retrospective study Wdowiak demonstrated myo-Ins effectiveness on PCOS women undergoing intracytoplasmic sperm injection (ICSI), concluding that 2 g myo-Ins plus 200 µg folic acid twice a day increased embryo development dynamics and accelerated blastocyst stage reaching time [134].

Further demonstrations of myo-Ins efficacy came from two other studies: the first is an IVF clinical trial on 133 PCOS women [135], in which the authors reported that supplementation with 1 g myo-Ins and 400 µg folic acid significantly augmented the number of mature oocytes compared to the control group, treated with folic acid and cyanocobalamin. In the second prospective, controlled, randomized trial by Özay and colleagues, the clinicians provided a supplement of 4 g myo-Ins and 400 µg folic acid to 98 infertile PCOS patients undergoing controlled ovarian hyperstimulation with rFSH and intrauterine insemination [136]. They observed that the myo-Ins treated group displayed a significant decrease in total rFSH dose required and cycle duration, as well as a higher pregnancy rate, when compared with controls.

The systematic review by Laganà et al. including eight RCTs and a total of 812 patients concluded that oral myo-Ins is able to reduce the amount of FSH in both PCOS and non-PCOS women undergoing IVF, while decreasing the length of controlled ovarian hyperstimulation only in PCOS patients [137].

Similarly, another systematic review confirmed the efficacy of myo-Ins supplementation in improving clinical pregnancy rate and reducing the units of rFSH in infertile non-PCOS patients undergoing ovulation induction for ICSI or IVF and embryo transfer [138].

Finally, a very recent double-blinded randomized controlled study by Mohammadi et al. provided evidence of myo-Ins efficacy in poor ovarian responders [139]. Indeed, myo-Ins supplementation in these patients significantly improved ART outcomes, such as fertilization rate, gonadotropin and ovarian sensitivity index, evidenced by a significant reduction in the required units of gonadotropin.

Overall, based on these findings, it could be interesting to make some pharmaco-economic considerations on the possible reduced costs related to assisted reproduction techniques, with myo-Ins supplementation and less gonadotropin use, before or during these procedures.

### 3.3. D-chiro-Inositol Dual Effects (Reduction in Estrogens in Estrogen-Sensitive Female Disorders; Increase in Androgens in Male Hypogonadism)

The discovery that D-chiro-Ins modulates the expression of the aromatase enzyme, influencing steroidogenesis and consequently the androgen/estrogen balance [31], led to the consideration that D-chiro-Ins supplementation may be a valid approach for male and female clinical conditions that would benefit from androgen increase and/or estrogen decrease [128,140] (Figure 4).

Interestingly, the study by Monastra et al. [141] showed that supplementation with 1 g/day D-chiro-Ins for 1 month to male volunteers with altered glycemia and/or hormonal status reduced serum estrone and estradiol levels (−85.0% and −14.4% respectively) and increased testosterone and dehydroepiandrosterone (+23.4% and +13.8% respectively). Besides normalizing the hormonal balance, the treatment with D-chiro-Ins decreased glycemia, insulinemia and HOMA index as well.

These results seem to confirm that D-chiro-Ins functions as an aromatase down-modulator, clearly opening new perspectives of research and therapeutic applications with this inositol.

Within the male population a potential target for D-chiro-Ins administration might be elderly men suffering from late-onset hypogonadism (LOH), who present with an impaired production of adequate levels of testosterone by testis and sperm cells, resulting in androgen deficiency [142,143]. Decrease in sexual activity, loss of body hair, subfertility and erectile dysfunction represent the main symptoms that these patients experience, followed by an inescapable decline in their quality of life.

Testosterone replacement therapy (TRT) is widely used for the treatment of LOH, although there is still an extensive debate whether it should be administered to men with this condition [142].

Importantly, in 2015, the FDA issued a warning about potential cardiovascular risks resulting from TRT. In addition, another concern derives from the fact that exogenous testosterone can suppress the hypothalamic-pituitary-gonadal axis through negative feedback, and TRT may lead to secondary spermatogenic failure and subsequent infertility [144].

Since they normalize testosterone to estradiol ratio and improve sperm concentration, motility and morphology, aromatase inhibitors (AIs) and selective estrogen receptor modulators (SERMs) could represent off-label options to TRT, especially for obese individuals or those at high risk of TRT [145]. However, these medications have not yet been established as common clinical practice.

Based on these premises and due to its proven safety [146], D-chiro-Ins represents a valuable alternative approach for these patients.

As such, encouraging results have been obtained from a recent pilot study by Nordio and colleagues. The authors reported that, after 1 month of a daily supplementation with 1800 mg D-chiro-Ins, 10 patients showed significantly increased serum testosterone and androstenedione levels. Conversely, estradiol and estrone levels were reduced, thereby providing an important demonstration that D-chiro-Ins behaved as a molecule that could affect aromatase activity. Furthermore, the treatment with D-chiro-Ins positively impacted on insulin resistance and waist circumference, improving patients’ sexual performance and physical strength [147].

Clearly, further studies with larger cohorts of patients should be encouraged in order to confirm these exciting data but such promising results should not be overlooked.

Notably, estrogen deficiency predisposes males to increased adiposity and metabolic dysfunction. Paradoxically, however, obesity in men has been associated with hyperestrogenism. Moreover, excessive estradiol stimulation has been postulated to play an exacerbating role in the progression of obesity and metabolic dysregulation [148]. Obese men are often characterized by low circulating androgens but elevated levels of circulating estrone and 17β-estradiol [149,150]. The reasons for this co-occurrence of obesity with hyperestrogenemia in men are not well defined but may include polymorphism in the aromatase gene CYP19A [151]. It has been proposed that increased peripheral aromatization of testosterone in obese men may enhance central estradiol signaling, suppressing gonadotropin production and contributing to a sustained state of hypogonadotropic hypogonadism [152].

From these observations, it becomes clear that obese men would greatly benefit from D-chiro-Ins supplementation, since this inositol optimizes glucose metabolism, reduces plasma insulin levels, and concomitantly, by modulating aromatase activity, promotes androgen production.

Additionally, in women, decreasing estrogen levels represents an interesting therapeutic target, particularly in the clinical management of uterine leiomyomas, also called fibroids. Indeed, estrogens, as well as progesterone, play a pivotal role in promoting cell proliferation and growth of myomas, that represent the most frequent form of benign neoplasia affecting female reproductive organs [153]. Although leiomyomas are mostly asymptomatic and tend to resolve spontaneously after menopause [154], some women may experiment severe symptoms, such as pelvic pain, dysmenorrhea and bleeding.

Unfortunately, up-to-date specific and safe treatments for myomas are still lacking, especially after ulipristal acetate (UPA) was withdrawn from the market in September 2020 due to the rare but serious side effect of liver failure [155].

On the other hand, off-label pharmacological treatments including progestogens, androgens, estrogen receptors antagonists, selective progesterone receptor modulators (SPRM) and gonadotropin-releasing hormone agonists (GnRHa) have been proven effective in reducing tumor size and symptoms before surgery or, possibly, to completely avoid the surgical procedure.

Furthermore, it is noteworthy that neoplastic tissues express high levels of the aromatase enzyme compared with the lower expression observed in non-neoplastic (normal) tissues [156]. In accordance, several groups reported that aromatase is significantly overexpressed in myoma cells compared with the adjacent normal myometrium [157,158], leading to a high level of in situ estrogens that may contribute to the growth advantage of myomas through an intracrine/autocrine mechanism [159]. Therefore, lowering estrogen levels could be clearly considered a valid therapeutic approach for fibroid management [160]. Indeed, aromatase inhibitors, like Letrozole, proved to be effective in reducing myoma size and volume, ameliorating patient symptoms and improving quality of life [161].

Since D-chiro-Ins affects aromatase expression, it can be a promising clinical option for managing uterine myomas. Montanino Oliva reported the case of two patients with leiomyomas associated with heavy menstrual bleeding, who sought pregnancy through ART [162]. These women were supplemented daily with a combination of Epigallocatechin gallate (EGCG), vitamin D and low dose of D-chiro-Ins for 3 months.

Several studies [163,164,165] had indeed demonstrated that natural compounds, like EGCG and vitamin D were effective in reducing myoma size and, in this contest, D-chiro-Ins may improve their efficacy in arresting myoma cell growth. Montanino Oliva observed a reduction in the fibroid volume of 73.8% and 68.4%. Moreover, a decrease in menstrual blood loss was recorded (−42.1% and −48.7%). Interestingly, 3 months after the end of the treatment, both patients underwent the ART procedure without the need for surgical intervention.

These results suggested a potentially high efficacy of this combination in reducing fibroid volume and menstrual bleeding, so that surgery could be avoided. D-chiro-Ins action on modulating aromatase activity might help to explain the important volume reduction reported in both cases, and it is tempting to speculate that vitamin D and EGCG exerted their anti-proliferative and proapoptotic effects, enhanced by the activity of D-chiro-Ins.

Certainly, these outstanding results need further confirmation on a larger sample of women.

Finally, D-chiro-Ins supplementation may be successfully used to decrease estrogen production and induce ovulation in anovulatory women [140]. In these patients, the first line treatment includes oral ovulation-inducing agents, such as the selective estrogen receptors modulator (SERM) clomiphene citrate, and Letrozole or Anastrozole, both aromatase inhibitors. These agents decrease estrogen biosynthesis and reduce the negative hypothalamic feedback on GnRH and FSH [166], but not without side effects. Indeed, especially for Letrozole, it seems to be contraindicated for the treatment of premenopausal women, since there is an increased risk of fetal cardiac and skeletal malformations associated with its administration [167].

Down-modulation in the expression of the aromatase enzyme by D-chiro-Ins, similar to the action of Letrozole, can be considered a valid approach to restore ovulation. However, because of its activity on aromatase and the subsequent androgen-rising effect, the baseline clinical condition of patients as well as the duration of supplementation should be carefully evaluated before starting therapy with D-chiro-Ins, in order to avoid a negative impact on the ovaries.

When hyperinsulinemic anovulatory women are supplemented with D-chiro-Ins, it restores ovulation, mainly functioning as an insulin-sensitizer by improving insulin signaling and decreasing the systemic hyperinsulinemia accordingly. Unfortunately, results on non-hyperinsulinemic patients are still lacking in the literature. 

A case report [168] showed that after six weeks of supplementation with D-chiro-Ins (1200 mg/day) ovulation occurred in two young non-PCOS lean women with anovulation. Indeed, in these patients, progesterone increased from 0.5 to 12 ng/mL, accompanied by endometrial thickening and menstruation around 50 days after the start of treatment.

Based on this finding and considering that the examined cases were normo-insulinemic, it is unlikely that insulin regulation played a pivotal part in restoring ovulatory function. The authors rather concluded that in such specific cases D-chiro-Ins probably acted mainly on aromatase expression, impairing estrogen biosynthesis and contributing to FSH release.

Hopefully, controlled studies with an appropriate sample of patients will be able to confirm this preliminary observation.

### 3.4. D-chiro-Inositol Supplementation: All a Matter of Dose and Time of Administration with a Focus on PCOS 

While scientific evidence indicates that myo-Ins and D-chiro-Ins can synergistically be integrated into the clinical management of PCOS, exerting therapeutic effects and representing a reliable alternative to conventional medications, the emerging role of D-chiro-Ins as an aromatase modulator and androgen-raising molecule clearly requires further reflection on its clinical use. In particular, when D-chiro-Ins is supplemented alone, the appropriate dosage and timing of treatment should be carefully evaluated and tailored to the different clinical pictures. Indeed, as previously discussed, following long-term or high-dose treatments, D-chiro-Ins could predominantly affect steroidogenesis increasing androgen levels and worsening patients’ clinical picture, especially in case of PCOS patients, already characterized by hyperandrogenism.

Accordingly, Bevilacqua and coworkers hypothesized that, like the aromatase inhibitor Letrozole, high doses of D-chiro-Ins administered to normal female mice would generate an androgenic PCOS-like model or other ovarian lesions [56].

To test this hypothesis, the authors supplemented wild type female mice with 250, 500 and 1000 mg/kg/day of D-chiro-Ins for 3 weeks. These doses provided approximate daily amounts of 5, 10 and 20 mg D-chiro-Ins/mouse, matching human doses of 1200, 2400 and 4800 mg/day respectively [169].

As a result, 250 mg/kg/day D-chiro-Ins contributed to the development of morphological features of human PCOS, similarly to those observed in mice treated with Letrozole, used as a positive control. Moreover, D-chiro-Ins-treated murine uteruses macroscopically resembled those in non-cycling animals and testosterone levels increased several-fold with respect to untreated negative control mice. Accordingly, a decrease in ovarian aromatase expression was recorded, providing the first evidence of a specific downregulation of aromatase mediated by D-chiro-Ins in an in vivo system and supporting the observations on cultured human granulosa cells from Sacchi [31].

The disruption of the ovarian organization constituted the principal result in mice treated with 500 and 1000 mg/kg/day D-chiro-Ins, while they exhibited minimal serum levels of testosterone and the ovarian content of aromatase was similar in both negative and positive controls. The authors speculated that these treatments could block the normal ovarian hormonal pathways, likely by inhibiting the expression/activity of cytochrome *P450scc* that catalyzes the first step of the steroidogenic cascade. Overall, these findings should encourage physicians to identify the most appropriate intervention with D-chiro-Ins, keeping in mind its dual function as well as patients’ metabolic and hormonal baseline conditions.

Indeed, as reiterated, D-chiro-Ins can improve some clinical conditions, but at the same time it can also worsen others. Those findings prompted Gambioli et al. to recommend specific daily doses and timing of D-chiro-Ins administration for several different medical conditions [128]. Of course, these observations and speculations need to be supported and confirmed hopefully through randomized, placebo-controlled, double-blind studies with greater sample sizes.

### 3.5. Enhancing Systemic Absorption of Inositols (Timing and Dose of Administration, Pharmaceutical Techniques)

Pharmacokinetic studies demonstrated that to optimize myo-Ins absorption, it should be supplemented twice a day, away from meals, at a dose of 2 g [170]. However, 25% to 75% of patients treated with myo-Ins may still be resistant to treatment [171]. These women are designated as “inositol resistant”. The etiology of the phenomenon of inositol resistance is not fully understood. In one study from Gerli et al., patients responding to myo-Ins supplementation showed significantly lower testosterone levels, higher SHBG and lower free androgen index [172], while in another study by Kamenov and colleagues most of the patients resistant to myo-Ins therapy were obese women [173]. Therefore, the state of obesity, the presence of insulin resistance, hyperandrogenism, dysbiosis or differences in compound bioavailability surely represent potential risk factors related to inositol resistance, since they could impair myo-Ins oral bioavailability.

Therefore, improving myo-Ins oral absorption represents one of the main challenges in clinical practice.

As reported for many drugs, encapsulation in soft gel capsules is a pharmaceutical technique currently used to foster the absorption of some compounds [174,175,176,177], compared with other conventional oral pharmaceutical forms.

Based on this evidence, myo-Ins was manufactured in the form of a soft gel capsule, resulting in improved gastrointestinal absorption that allows a reduction of the administered dose by one-third compared to the powdered form to achieve the same circulating inositol level, and at the same time overcoming the dose-dependent gastrointestinal side effects, thus improving patient compliance [170].

**Figure 7:**
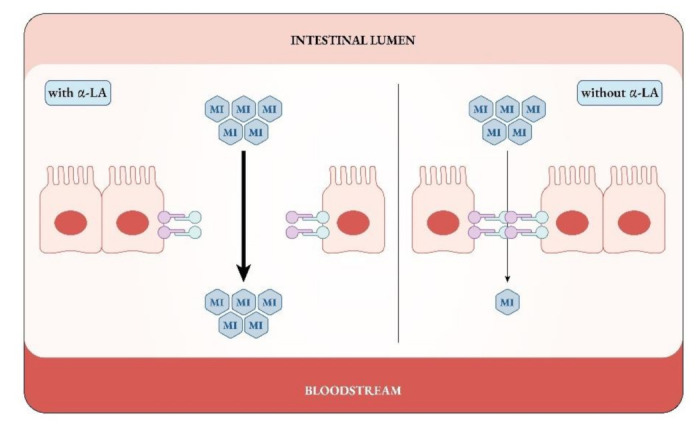
α-lactalbumin (α-LA) increases myo-inositol (MI) passage across intestinal cellular monolayer, causing a temporary opening of the tight junctions between the cells.

Recently, researchers proposed the concomitant administration of myo-Ins and the milk protein α-LA, already known for its properties as a nutrient as well as for enhancing the absorption of metal ions and vitamins [178,179]. A pharmacokinetic study from Monastra et al. on 18 healthy volunteers [180] provided evidence that the combination of myo-Ins plus α-LA guarantees a greater bioavailability of myo-Ins, compared to when myo-Ins is administered alone. As reported by the authors, the analysis of myo-Ins plasma concentrations measured with or without concomitant α-LA administration yielded interesting outcomes of pharmacokinetic parameters for the combined formulation. In support of the efficacy of this combined treatment, in 2018 the study by Montanino Oliva and colleagues found that that 23 of the 37 women (62%) ovulated following myo-Ins treatment, while 14 (38%) were identified as resistant. As such, in this group myo-Ins plasma levels did not increase. When these myo-Ins-resistant patients received the combined treatment with myo-Ins and α-LA, 86% of them ovulated and their myo-Ins plasma levels were found to be significantly higher than the baseline values [181]. Their lipidic and hormonal profiles improved as well. In the same year, the mechanism behind this effect was elucidated. Monastra et al. discovered that, in presence of α-LA, myo-Ins increased its passage across the Caco-2 cell monolayer, used as a standard in vitro model of intestinal epithelium [182]. Specifically, the presence of α-LA triggered a transient opening of the tight junction between the cells, allowing to myo-Ins to be transported passively across this cell layer (Figure 7). The same effect, due to α-LA, was found also with D-chiro-Ins [183].

A few years later, aiming to enlarge and further explore the results obtained by Montanino Oliva [181], Hernandez-Marin et al. [184] carried out a multicenter clinical study comparing the efficacy of the combination of myo-Ins with α-LA in two groups of PCOS women from Mexico and from Italy. After 3 months of supplementation all patients experienced a general improvement, which was maintained at 6 months. In fact, BMI decreased, progesterone levels significantly increased, and glucose, insulin, FSH and LH improved as well, even if not always significantly. 

Taken together these results confirm that myo-Ins oral absorption represents a limiting factor for the successful treatment of PCOS but through the use of various measures this issue can be overcome.

### 3.6. The Consequences of Inositol Deficiency Due to Pharmacological Treatments, Malabsorption or Competition with Dietary Glucose

It should also be stressed that, when high doses of D-chiro-Ins or other sugar-like molecules are supplemented concomitantly with myo-Ins, they seem to negatively impact on its availability in the human body. As a matter of fact, Garzon et al. observed that D-chiro-Ins, sorbitol and maltodextrin, when provided together with myo-Ins, significantly decreased its absorption and plasma concentration, compared to when myo-Ins was supplemented alone [7]. As a possible explanation, the authors postulated a competitive action among these molecules at inositol transporters, mainly SMIT2. Indeed, this protein exhibits similar affinity for both inositol stereoisomers, as demonstrated by the K_m_ values (120–150 µM for myo-Ins and 110–130 µM for D-chiro-Ins). However, considering that under physiological conditions the serum concentration of D-chiro-Ins is less than 100 nM, it is unlikely that it can interfere with myo-Ins absorption, since serum concentrations of myo-Ins typically range from 26.8 to 43.0 µM, which is significantly higher [185,186]. The same shall apply to the formulations in which myo-Ins and D-chiro-Ins are combined in a proper ratio, such as the 40:1 formula.

On the contrary, when D-chiro-Ins or other sugars from food are consumed at high dosage (≥1 g) and/or concomitantly with myo-Ins, a strong competition for SMIT2 transporters may occur, decreasing myo-Ins absorption in the gut and consequently altering plasma myo-Ins/D-chiro-Ins ratio that possibly accounts for pathological conditions.

In addition, it should not be underestimated that some pharmacological treatments, particularly antiepileptic drugs (AEDs), such as sodium valproate (VA), and mood stabilizers for bipolar disorder, such as lithium (Li^+^), can cause myo-Ins depletion. All of these medications share the “inositol-depletion hypothesis”, put forward to explain their therapeutic mechanism [187]. According to this theory, Li^+^ acts principally by inhibiting the monophosphatase (IMPase) and the inositol polyphosphatase (IPP), key enzymes in inositol synthesis, while VA inhibits the myoinositol-phosphate synthase (MIPS).

Although these drugs superficially appear beneficial to patients, since they are able to control seizures and mood disbalances, their chronic administration is not completely safe and may expose patients to side effects mostly associated with inositol depletion in peripheral tissues. Among these effects are hypothyroidism, weight gain, hyperinsulinemia, dyslipidemia, impairment of kidney function and adverse dermatological effects that are quite commonly reported [188,189,190]. Noteworthy, PCOS is one of the most serious side-effects reported by AEDs-treated women [190,191,192,193], who experience reduced estradiol and progesterone and increased testosterone, leading to hypogonadism, amenorrhea or oligomenorrhea, along with sexual dysfunction and infertility [194].

Importantly, even though these conditions may sometimes spontaneously resolve after a few weeks of treatment, or revert to baseline with drug discontinuation, they certainly worsen patients’ quality of life, weakening their compliance to therapeutic protocols.

Several scientific studies have documented myo-Ins supplementation as a safe and efficient approach in treating PCOS symptoms, improving hormonal profile, hyperandrogenism, menstrual cycle, oocyte quality and psychological disturbances [54,195]. Moreover, myo-Ins in combination with Selenium, has been reported to be effective in restoring euthyroidism in patients with subclinical hypothyroidism or autoimmune thyroiditis [196]. Therefore, supplementing with this inositol could be a valid option to counteract the peripherical side effects connected to bipolar disorder and epileptic treatment. Clearly, the dose of myo-Ins supplemented should be properly settled so as not to increase its levels in the brain and interfere with the central pharmacological therapeutic effect. Some studies reported a myo-Ins dose of 3–6 g/day could effectively promote recovery from the peripheral adverse effects without passing the blood brain barrier and hindering the beneficial central action of pharmacological treatments [197,198]. Moreover, since D-chiro-Ins is obtained from myo-Ins, it should be considered that drug-induced depletion of the latter also influences D-chiro-Ins levels. As such, administering the combination of myo-Ins and D-chiro-Ins seems to be therapeutically more effective in respect to supplementing myo-Ins alone. Indeed, even if myo-Ins is naturally converted to D-chiro-Ins, supplementing these two isomers in combination will enable the rebalancing of inositol depletion and its consequences more efficiently and in a shorter time.

The possible ratio between myo-Ins and D-chiro-Ins may physiologically range from 10:1 to 100:1 and the ratio 80:1 in favor of myo-Ins is particularly encouraged [190].

From these premises, the concomitant supplementation with inositol to patients in therapy with Li^+^ or AEDs could represent an intriguing proposal. Interestingly, this combined treatment would safely reduce the peripheral side effects, ameliorating patients’ compliance and improving quality of life.

## 4. Conclusions

Over time the scientific vision of inositols has been through an important evolution, allowing the realization that these isomers, although quite similar from a chemical point of view, actually play several different roles, at times even opposing functions.

During these past twenty years of research on inositols we learned a lot: namely that a proper myo-Ins to D-chiro-Ins ratio governs the healthy state of organs and tissues, while an imbalance in inositol levels or their peripheral tissue depletion may account for pathological conditions. Therefore, restoring the inositol physiological ratio or altering this ratio in a controlled manner are proving to be two reasonable aims to achieve specific effects.

Consolidated findings have provided convincing evidence on the therapeutic efficacy of inositols in PCOS treatment and to support both female and male reproduction, as well as on myo-Ins’s effectiveness in preventing GDM and NTDs.

Despite evidence, the validity of the myo-Ins to D-chiro-Ins 40:1 ratio should be further elucidated and supported by large-scale clinical trials as well as pharmacokinetic studies.

Moreover, the newly discovered role of D-chiro-Ins as an aromatase modulator has imposed the need to reflect on the proper use of this isomer in therapy, since D-chiro-Ins may influence steroidogenesis and consequentially alter the estrogen/androgen ratio in favor of androgens. Thus, the use of D-chiro-Ins in clinical practice should be tailored to the baseline features of patients for whom it is intended. Clearly, PCOS women who already experience hyperandrogenism would not benefit from isolated D-chiro-Ins treatment, especially as long-term supplementation and at high doses. Conversely, D-chiro-Ins would be useful for those patients who could benefit from an increase in androgen levels at the expense of estrogens.

Clearly there is still a lot to understand and learn about inositols, which over the years have tiptoed to come alongside the classic pharmacological treatments, gaining a prestigious role for their efficacy, safety and compliance.

## Figures and Tables

**Table 1:** Myo-Ins (MI) to D-chiro-Ins (DCI) ratios in different tissues, both in physiological and insulin resistance conditions. Data were retrieved and adapted from the references [18,30,32].

	Physiological Conditions		Insulin Resistance Conditions	
	MI (%)	DCI (%)	MI (%)	DCI (%)
Fat	65	35	98	2
Liver	70	30	99.3	0.7
Muscle	74	26	98.1	1.9
Blood	97	3	99.6	0.4
Kidney	98	2	98.2	1.8
Intestine	98	2	98.2	1.8
Spleen	98.8	1.2	99	1
Heart	99.5	0.5	99.3	0.7
Brain	99.5	0.5	99.2	0.8
Follicular fluid	99.02	0.98	15	85
Ovary (Theca)	95.24	4.76	16.67	83.33

## Data Availability

Not applicable.

## Abbreviations

AEDs	Antiepileptic drugs
AIs	Aromatase inhibitors
AMH	Anti-Mullerian hormone
ART	Assisted Reproductive Technology
BBB	Blood brain barrier
BMI	Body mass index
D-chiro-Ins	D-chiro-inositol
E2	Estradiol
EGCG	Epigallocatechin gallate
FF	Follicular fluid
FSH	Follicle-Stimulating Hormone
G6P	Glucose-6-phosphate
GDM	Gestational Diabetes Mellitus
ICSI	Intracytoplasmic sperm injection
IMPA-1	Inositol monophosphatase-1
IMPase	Inositol monophosphatase-1
InsP2	Inositol-bisphosphate
InsP3	Inositol-3-phosphate
IVF	In Vitro Fertilization
LH	Luteinizing Hormone
MIPS	myoinositol-phosphate synthase
myo-Ins	myo-inositol
NTDs	Neural Tube Defects
OAT	Oligoasthenoteratozoospermia
PCOS	Polycystic Ovary Syndrome
SBGH	Sex Binding Globulin Hormone
SERMs	Selective Estrogen Receptor Modulators
SMIT	Sodium/myo-inositol transporter
TRT	Testosterone Replacement Therapy
UPA	Ulipristal acetate
VA	Valproic acid
α-LA	α-lactalbumin
